# SARS-CoV-2 Positive Serology and Islet Autoantibodies in Newly Diagnosed Pediatric Cases of Type 1 Diabetes Mellitus: A Single-Center Cohort Study

**DOI:** 10.3390/ijms24108885

**Published:** 2023-05-17

**Authors:** Anca Andreea Boboc, Carmen Nicoleta Novac, Alexandra Gabriela Marin, Mara Ioana Ieșanu, Cristina Plătică, Teodora Buzescu, Maria Teodora Coșoreanu, Felicia Galoș

**Affiliations:** 1 Department of Pediatrics, Carol Davila University of Medicine and Pharmacy, 020021 Bucharest, Romania; anca.orzan@umfcd.ro (A.A.B.); dr.carmen.novac@gmail.com (C.N.N.); felicia.galos@umfcd.ro (F.G.); 2 Department of Pediatrics, Marie Curie Emergency Children’s Hospital, 041451 Bucharest, Romania; mara-ioana.iesanu@drd.umfcd.ro (M.I.I.); cristina.platica@yahoo.com (C.P.); tomoiuteodora@gmail.com (T.B.); 3 Department of Infectious Diseases, Prof. Dr. Matei Balș National Institute of Infectious Diseases, 021105 Bucharest, Romania; alexandra-gabriela.n.marin@rez.umfcd.ro; 4 Department of Physiology, Carol Davila University of Medicine and Pharmacy, 020021 Bucharest, Romania

**Keywords:** type 1 diabetes mellitus, SARS-CoV-2 infection, SARS-CoV-2 antibodies, islet autoantibodies, IA-2A, diabetic ketoacidosis, C-peptide

## Abstract

Acute respiratory syndrome coronavirus 2 (SARS-CoV-2) infection, although presenting less severe forms of the disease in children, seems to play a role in the development of other conditions, including type 1 diabetes mellitus (T1DM). After the beginning of the pandemic, an increase in the number of T1DM pediatric patients was observed in several countries, thus leading to many questions about the complex relationship between SARS-CoV-2 infection and T1DM. Our study aimed to highlight possible correlations between SARS-CoV-2 serology and T1DM onset. Therefore, we performed an observational retrospective cohort study that included 158 children diagnosed with T1DM in the period April 2021–April 2022. The presence or absence of SARS-CoV-2 and T1DM-specific antibodies and other laboratory findings were assessed. In the group of patients with positive SARS-CoV-2 serology, a higher percentage had detectable IA-2A antibodies, more children were positive for all three islet autoantibodies determined (GADA, ICA, and IA-2A), and a higher mean HbA1c value was found. No difference existed between the two groups regarding DKA presence and severity. A lower C-peptide level was found in the patients presenting diabetic ketoacidosis (DKA) at T1DM onset. When compared to a group of patients diagnosed before the pandemic, an increased incidence of both DKA and severe DKA, as well as a higher age at diagnosis and higher levels of HbA1c were present in our study group. These findings have important implications for the ongoing monitoring and management of children with T1DM after the COVID-19 pandemic and highlight the need for further research to better understand the complex relationship between SARS-CoV-2 infection and T1DM.

## 1. Introduction

Type 1 diabetes mellitus (T1DM) represents one of the most common chronic diseases affecting children with a major impact on the quality of life of patients and parents and numerous possible long-term complications. The autoimmune pathogenesis of T1DM, leading to the destruction of pancreatic beta-cells, results from a complex interaction between genetic factors and environmental triggers such as various viruses, microbiota, diet, and vitamin D deficiency [1,2,3]. The long-known involvement of numerous viral agents in the development of T1DM has led to an increased interest in analyzing the implications of the coronavirus disease 2019 (COVID-19) pandemic on the incidence of T1DM in children [4,5,6]. Acute respiratory syndrome coronavirus 2 (SARS-CoV-2) infection generally showed a less severe evolution during the acute phase in children compared to that seen in the adult population [7]. In spite of this fact, the influence of SARS-CoV-2 on the function of the immune system in children is characterized by a complexity that is yet to be completely understood. In addition to COVID-19-associated multi-system inflammatory syndrome in children (MIS-C), the most important serious complication developed by children after the initial infection, the pediatric population showed a higher risk of developing several other diseases with autoimmune background after the beginning of the COVID-19 pandemic [8,9,10,11]. The differences in the evolution of SARS-CoV-2 infection in children compared to adults reside in the specific characteristics of B cell and T cell immune responses in children [12]. The hyperinflammatory status induced by SARS-CoV-2 infection is able to trigger several immune pathways leading to autoimmune reactions [11,13].

Multiple studies have reported a significant increase in the number and severity of T1DM new cases after the beginning of the COVID-19 pandemic [5,6,14,15,16,17,18]. In our center, the total number of new T1DM cases increased by 30.08% between February 2020 and March 2021, compared to the previous year [19]. Moreover, the proportion of children presenting with diabetic ketoacidosis (DKA) at the time of diagnosis was 67.4% higher during the pandemic [20]. One of the objectives of this study was to determine whether this increase in the DKA presence and severity at T1DM onset persisted in the period April 2021–April 2022. In order to further analyze the complex relationship between SARS-CoV-2 infection and T1DM, we determined the presence of SARS-CoV-2 antibodies in patients newly diagnosed with T1DM in our clinic. One of the aims of this study was to determine the differences existing between the groups of patients with positive and negative SARS-CoV-2 serology.

We evaluated the correlations between the presence of SARS-CoV-2 antibodies and the T1DM-associated autoantibodies, i.e., islet cell autoantibodies (ICA), glutamic acid decarboxylase antibodies (GADA), and protein tyrosine phosphatase 2 antibodies (IA-2A). Islet autoantibodies, as markers of the autoimmune mechanisms involved in the pathogenesis of T1DM, have shown utility in T1DM diagnosis and in the prediction of the disease in the pre-symptomatic period [20,21,22]. Other parameters analyzed were C-peptide levels, as a marker of residual insulin secretion, and glycosylated hemoglobin (HbA1c) values that reflect the blood glucose levels over the past 3 months [23,24].

DKA represents an important metabolic complication of T1DM, resulting from persistent insulin deficiency, and has a possible life-threatening evolution [25]. We divided the study sample according to the presence or absence of DKA at T1DM, and we evaluated the characteristics of these two groups of patients.

The main goals of the study were to find the possible differences regarding the characteristics of newly diagnosed T1DM with positive and negative SARS-CoV-2 serology and to assess the changes that occurred during the pandemic in the characteristics of patients and disease.

## 2. Results

### 2.1. Demographic and General Characteristics

A total of 158 pediatric patients were diagnosed with T1DM in our center during the period April 2021–April 2022. The study population included 83 (52.5%) female patients. The median age of diagnosis was 9 years old, IQR = [5.00–12.00]. The most frequently represented age group was 6–12 years old with 69 (43.7%) patients, while 60 (37.9%) of the patients were 0–6 years old, and 29 (18.4%) of the children belonged to the age group 12–18 years. A total of 103 (65.2%) patients were living in an urban area. Fourteen (8.9%) children presented a positive family history of T1DM.

### 2.2. Laboratory Findings

Positive serology for SARS-CoV-2 was present in 37 (23.3%) patients. Four (10.8%) of these children developed only IgM SARS-CoV-2 antibodies, while twenty-six (70.3%) patients presented only IgG SARS-CoV-2 antibodies, and seven (18.9%) patients tested positive for both types of antibodies. Among these patients, 10 had a known and documented history of SARS-CoV-2 infection.

At least 1 positive beta-cell autoantibody was detected in 132 (83.5%) children, and 41 (31%) patients were positive for all 3 tested autoantibodies. Positive values for GADA, ICA, and IA2A were detected in 106 (69.7%), 81 (53.3%), and 83 (55.7%) of the cases, respectively.

DKA was present in the majority of children (100, 67.1%). The severe form (pH < 7.1) of DKA was found in 31 (31%) patients, the moderate form (pH < 7.2) in 38 (38%) cases, and the mild form (pH < 7.3) in 31 (31%) cases. The median pH value was 7.25 IQR [7.15–7.40]. HbA1c had a mean value of 12.14% ± 1.96%. The median value of the C-peptide was 0.58 ng/mL IQR [0.39–1.01]. 

Anti-thyroid peroxidase antibodies were found in 22 (14.5%) patients, and 15 (9.4%) patients presented positive IgA or IgG anti-tissue transglutaminase antibodies.

The study population presented a median vitamin D value of 24.70 ng/mL IQR [15.90–30.35]. Vitamin D deficiency (vitamin D < 20 ng/mL) was identified in 50 (35.5%) patients.

### 2.3. Differences between the Patients with Positive and Negative SARS-CoV-2 Antibodies

**Figure 1:**
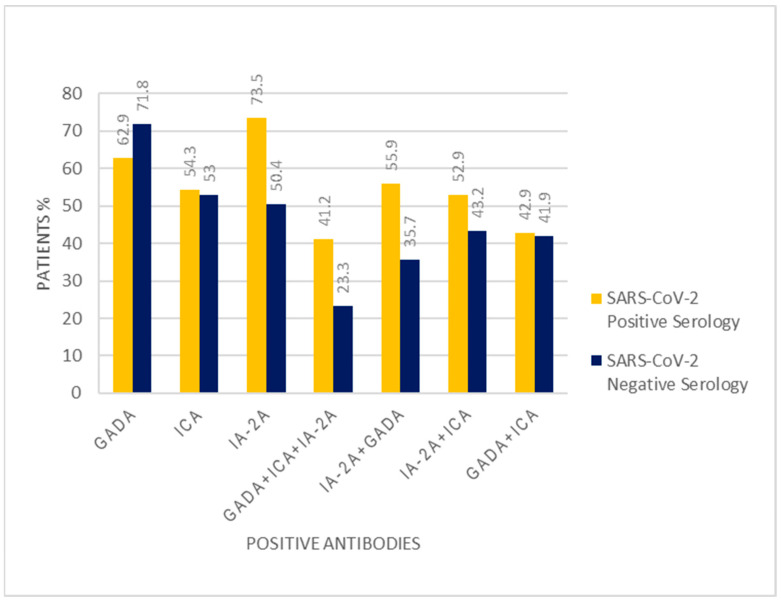
T1DM-specific antibody positivity rates in the groups of patients with positive and negative SARS-CoV-2 serology.

In the group of patients with SARS-CoV-2 positive serology, males were significantly more represented (56.8% vs. 44.6%, *p* < 0.001). The background of most of the patients was urban in both groups (59.5% vs. 66.9%, *p* = 0.40). There was no significant difference in the age of diagnosis between the group of patients with positive SARS-CoV-2 serology and the group with negative SARS-CoV-2 serology (8.18 years vs. 8.53, *p* = 0.63). The pH value at diagnosis did not differ significantly between the two groups of patients (7.26, IQR (7.11–7.40) vs. 7.25, IQR (7.17–7.40), *p* = 0.73). The percentage of patients presenting with DKA was higher in the group with positive SARS-CoV-2 antibodies, but the difference was not statistically significant (72.2% vs. 65.5%, *p* = 0.45). No difference was found between the two groups in the percentage of children presenting with the severe form of DKA (22.2% vs. 20.4%, *p* = 0.81). The mean HbA1c value was higher in the group of patients with positive SARS-CoV-2 serology (12.96% vs. 11.88%, *p* = 0.004). Vitamin D levels were similar in both groups (23.30, IQR 15.30–30.02 ng/mL vs. 25.10, IQR 15.75–30.40 ng/mL, *p* = 0.75). C-peptide values did not differ significantly between the two groups of patients (0.58, IQR 0.42–1.02 ng/mL vs. 0.57, IQR 0.37–1.01 ng/mL, *p* = 0.82). The percentages of patients with positive GAD and patients with positive ICA antibodies did not differ significantly between the two groups (62.9% vs. 71.8%, *p* = 0.31 and 54.3% vs. 53.0%, *p* = 0.89, respectively). A higher percentage of patients tested positive for IA-2A antibodies in the group with positive SARS-CoV-2 serology (73.5% vs. 50.4%, *p* = 0.01). More patients presented positive serology for all three types of tested islet autoantibodies in the group with positive SARS-CoV-2 antibodies (41.2% vs. 23.3%, *p* = 0.03). A higher percentage of children from the group with positive SARS-CoV-2 serology developed both GADA and IA-2A (55.9% vs. 35.7%, *p* = 0.03). Additionally, positivity for both ICA and IA-2A was detected more often in the group of children with positive SARS-CoV-2 antibodies (52.9% vs. 32.2%, *p* = 0.02). No significant difference was found between the two groups in the proportion of positive serology for both GADA and ICA (42.9% vs. 41.9%, *p* = 0.91). (Table 1, Figure 1).

### 2.4. Differences between the Patients with and without DKA at T1DM Onset

When we grouped the patients based on the presence or absence of a DKA diagnosis at T1DM onset, a lower age at diagnosis was found in the first group (7 years (4.0–11.0) vs. 11 years (7.5–12.0), *p* = 0.04). No significant differences were found in the sex and background distribution (M/F: ratio 1.00 vs. 0.88, *p* = 0.72; rural/urban ratio: 0.44 vs. 0.75, *p* = 0.15). SARS-CoV-2 antibodies were present in similar percentages in both groups (26.0% and 20.4%, *p* = 0.45). The mean HbA1c value was similar in both groups (12.34% ± 1.89% vs. 11.92% ± 2.13%, *p* = 0.22). No differences in the GADA, ICA, and IA-2A positivity rates were identified (68.8% vs. 70.8%, *p* = 0.79; 56.3% vs. 47.9%, *p* = 0.34; 61.3% vs. 45.8%, *p* = 0.08, respectively). There were no significant differences between the two groups in the percentages of patients who tested positive for all three antibodies (26.6% vs. 27.1%, *p* = 0.95), for GADA and ICA (41.7% vs. 41.7%, *p* = 1.00), for GADA and IA-2A (44.1% vs. 33.3%, *p* = 0.21), or for ICA and IA-2A (38.7% vs. 66.7%, *p* = 0.53). The values of vitamin D did not significantly differ between the two groups (23.95, IQR 14.37–30.37 ng/mL vs. 25.80, IQR 17.25–30.45 ng/mL, *p* = 0.57). C-peptide levels were lower in the group of patients presenting with DKA (0.49, IQR 0.33–0.85 ng/mL vs. 0.78, IQR 0.51–1.16 ng/mL, *p* < 0.001) (Table 2).

### 2.5. Differences between the Groups of Patients Diagnosed in April 2021–April 2022 vs. March 2020–February 2021 vs. before the Pandemic

**Figure 2:**
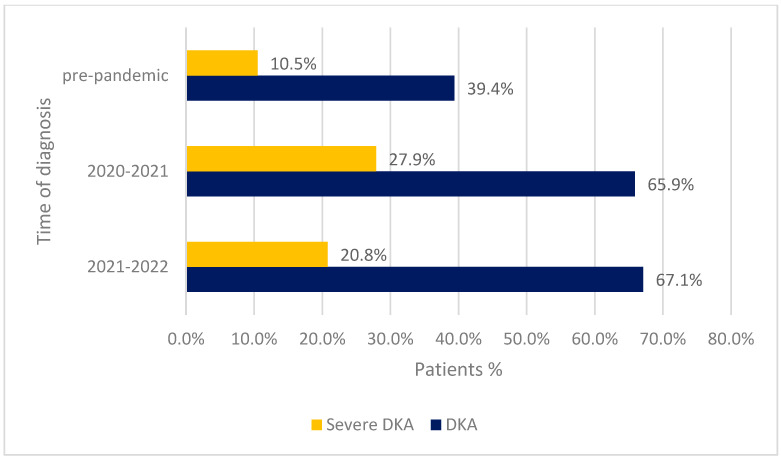
The frequencies of DKA diagnosis and DKA severe form according to the time of diagnosis.

There were no differences between the groups of patients diagnosed in the period April 2021–April 2022 (2021 group), those diagnosed between March 2020 and February 2021 (2020 group), and those diagnosed before the pandemic (the pre-pandemic group), regarding the sex distribution (M/F ratio 0.9 vs. 1.04, *p* = 0.53 and 0.9 vs. 1.19, *p*= 0.15, respectively). The rural background was better represented in the 2021 group than in the pre-pandemic group (34.8% vs. 16.0%, *p* < 0.001), without any significant difference between the 2021 group and the 2020 group (34.8% vs. 38.1%, *p* = 0.55). The age at the time of diagnosis was higher in the 2021 group compared to both the other groups (9.00 (5.00–12.00) vs. 7.20 (7.07–7.30) and 9.00 (5.00–12.00) vs. 7.00 (3.00–10.00), *p* values <0.01). The diagnosis of DKA was more frequent in the 2021 group than in the pre-pandemic group (67.1% vs. 39.4%, *p* < 0.001). There was no significant difference in the frequency of DKA between the two pandemic groups (67.1% vs. 65.9%, *p* = 0.83). In the 2021 group, a significantly higher percentage of children developed the severe form of DKA compared to the pre-pandemic group (20.8% vs. 10.5%, *p* = 0.002). Similar frequencies of severe DKA were identified in both the 2021 group and the 2020 group (20.8% vs. 27.9%, *p* = 0.15). Mean HbA1c levels were higher in the 2021 group than in the pre-pandemic group (12.14% ± 1.96% vs. 11.32% ± 2.18%, *p* < 0.001), with similar mean HbA1c levels being found in the 2020 group (12.14% ± 1.96% vs. 12.47% ± 2.19, *p* = 0.18) (Table 3, Figure 2).

## 3. Discussion

It has been 3 years since the COVID-19 pandemic became the center of medical research, and the scientific community is still trying to understand all the consequences it has presented. By interfering with the functioning of fine-regulated immune pathways, SARS-CoV-2 leads to a state of increased susceptibility for developing autoimmune-mediated diseases [11]. Moreover, a series of changes in the epidemiology of multi-factorial determined diseases emerged from the disruption of the classical circulation and occurrence of other viral and bacterial infections because of the isolation during the lockdown period and the emergence of a new infection with an increased incidence [26,27]. T1DM, a condition with a high prevalence in the pediatric population, involves an autoimmune-mediated process of destruction of insulin-producing pancreatic beta-cells. The most important markers of the autoimmune pathogenic mechanisms are represented by islet autoantibodies. Therefore, we investigated whether a history of SARS-CoV-2 infection, proven by positive specific serology, presents any correlations with the levels of islet autoantibodies of newly diagnosed patients. As asymptomatic forms are frequent in children, and because we could not be certain that a number of infections were not absent because of insufficient COVID-19 testing in some cases, the levels of SARS-CoV-2 antibodies represent the only objective way of identifying a former SARS-CoV-2 infection, and they also have the advantage of being easily available. On the other hand, exploring SARS-CoV-2 serology presents the disadvantage of being influenced by many factors, the most important of which being the amount of time that has passed from the infection. Therefore, the findings should be looked at with caution.

The positivity rates of GADA, ICA, and IA-2A were 69.7%, 53.3%, and 55.7%, respectively. The percentages of children with positive islet antibodies were lower than those detected in a study including patients with T1DM from Japan, in which GADA was detected in 80% of the patients, and IA-2A was detected in 60% [28]. In another study, 56% of the newly diagnosed children tested positive for GADA, and 63% tested positive for IA-2A [21]. The high percentage of patients that were positive for all three antibodies was to be expected considering that the total number of antibodies is a stronger predictive factor of the evolution towards diabetes than a single antibody, and all of the patients included had a certain T1DM diagnosis [22].

The percentage of 23.3%, representing the children with positive SARS-CoV-2 serology, is higher than that found in a study conducted in Colorado, in which SARS-CoV-2 antibodies were detected in only 0.8% of the patients with newly diagnosed T1DM [29]. In another study from 2021 including healthy children aged 1–18 years old from Colorado and children aged 1–10.9 years old from Bavaria, 32.3% and 0.61% of the patients, respectively, presented positive SARS-CoV-2 antibodies [30]. In the same study, the presence of T1DM-specific antibodies was assessed, and no correlations were found between the presence of islet autoantibodies and a positive serology for SARS-CoV-2 [30].

In the children who tested positive for SARS-CoV-2 antibodies, a higher frequency of positive serology was found for IA-2A (73.5% vs. 50.4%, *p* = 0.01). Moreover, in the group with positive SARS-CoV-2 serology, more patients developed all three tested islet autoantibodies. The percentage of patients that developed only IA-2A + GADA or IA-2A + ICA was also significantly higher in this group of patients. There were no differences between the groups regarding the proportion of positive serology for the other two types of T1DM antibodies, neither individually nor combined. Within the limits of using serological markers and not a more accurate test detecting a contemporary SARS-CoV-2 infection, these results may support the idea that SARS-CoV-2 infection is able to promote autoimmune processes, including those involved in the development of T1DM. The correlation of SARS-CoV-2 positive serology only with IA-2A and not with the other antibodies, when considered individually, represents an important finding considering the fact that the detection of IA-2A seems to be more predictive for the clinical onset of T1DM than other types of islet autoantibodies [31]. An Italian study including 532 patients diagnosed with T1DM over a period of almost 30 years showed that IA-2A positivity is correlated with the presence of DKA at T1DM onset [32]. However, it is difficult to estimate the relationship between SARS-CoV-2 infection and the development of these antibodies without knowing the evolution over time of the levels of islet autoantibodies in these children. In reaching more accurate conclusions it would have been of great utility to know if the levels of islet autoantibodies showed a significant increase after the moment when the patients were affected by a SARS-CoV-2 infection. Moreover, it would be important to set apart and further investigate the possible group of patients with negative islet autoantibodies before the moment of SARS-CoV-2 infection who would, later on, develop these markers of autoimmunity.

There was no significant difference in DKA presence and severity between the groups of patients with positive and negative SARS-CoV-2 serology. Considering the fact that several studies, including one conducted in our clinic, reported an increase in DKA incidence and severity after the beginning of the pandemic, a higher percentage of this complication could have been expected in children with positive SARS-CoV-2 antibodies [5,6,14,15,16,17,18]. An explanation for this finding could reside from the fact that we cannot be absolutely certain that children with negative SARS-CoV-2 antibodies did not experience a SARS-CoV-2 infection at some point in their history without it leaving a persistent serological mark, but being able to determine alterations of the islet cells functioning or of the immune processes. From another point of view, perhaps other factors with a possible influence on DKA incidence and severity should be taken into consideration, including phenomena described during the pandemic such as the alteration in the circulation of other viruses, including those with a known implication in the development of T1DM [26,27]. The increased level of stress represents another factor present during the pandemic that has possible implications in the development of T1DM [33,34]. On the other hand, the existing data show that SARS-CoV-2 is able to directly induce β-cell damage (without it representing a common event) and is also able to determine immune regulatory alterations involved in the development of autoimmune diseases, including T1DM [13,35]. However, it is not clear to what extent these processes can actually lead to the onset of T1DM [13,35].

The patients presenting DKA at T1DM onset had a smaller mean age at diagnosis and lower C-peptide levels, without any significant differences in the positivity rates of SARS-CoV-2 antibodies or islet autoantibodies. The presence of DKA is a consequence of significant alterations in the metabolic pathways that can be determined by a more reduced residual insulin production indicated by the lower C-peptide levels [23,36]. Other studies also found a correlation between DKA, lower C-peptide values, and higher HbA1c levels [36,37,38,39].

When we compared the group of patients diagnosed in the period April 2021–April 2022 to the group of patients diagnosed with T1DM in our clinic before the pandemic, a higher incidence of DKA was identified, as well as a higher percentage of children diagnosed with severe DKA. Moreover, there were no significant differences concerning DKA presence and severity between this group of patients and that diagnosed in the period February 2020–March 2021. These findings are giving our results consistency and persistence. The only significant difference existing between the two groups of patients diagnosed during the pandemic was the higher age of diagnosis identified in the patients from the 2021 group.

The main limitations of this study were represented by the reduced size of the study group and by the fact that not all the assessed variables were available for all the patients. Furthermore, autoantibodies for zinc transport 8 and insulin, the two other major autoimmune markers of T1DM, usually studied together with GADA, ICA, and IA-2A, are not routinely determined in our hospital, but assessing their values and correlations would have had a great utility. Another limitation of our study was represented by an increased risk that type I errors could have appeared, given the fact that several univariate analyses were performed on the same set of data.

Taking into consideration the fact that the levels of SARS-CoV-2 antibodies express a decreasing evolution as time passes from the moment of infection, the percentage of children who presented a SARS-CoV-2 infection at some point in the past could be different than the percentage of patients with positive SARS-CoV-2 antibodies detected at the onset of T1DM. This aspect could lead to a difference between the characteristics we found for the patients with positive SARS-CoV-2 serology and those possessed by the total group of children that developed SARS-CoV-2 infection in their history.

Further prospective cohort studies analyzing the evolution over time of T1DM-specific antibodies and the development of T1DM in children with a known history of SARS-CoV-2 infection are necessary in order to obtain some clarity over the complex relationship between COVID-19 and T1DM. Further research is needed to better understand the mechanisms that link SARS-CoV-2 infection and T1DM, focusing on the involvement of specific cytokines and immune pathways. Furthermore, it is necessary to assess the evolution of children diagnosed with T1DM during the pandemic, monitoring the degree of glycemic control; the development of T1DM complications; and the association over time of other autoimmune pathologies. Given the increase in the frequency and severity of DKA, a metabolic complication with a complex evolution and a possible fatal outcome, it is highly necessary to take more measures in order to prevent the development of DKA, including increasing awareness about T1DM symptoms in the general population and implementing screening methods for T1DM in the pediatric population. Finally, it is important to consider the benefits of childhood vaccination against SARS-CoV-2 to children with T1DM.

## 4. Materials and Methods

### 4.1. Study Population

The performed study was an observational retrospective cohort study. In order to assess the characteristics of pediatric patients who developed T1DM after the beginning of the COVID-19 pandemic, we included patients newly diagnosed with T1DM at Marie Curie Emergency Children’s Hospital in Bucharest during a period of 13 months (1 April 2021–30 April 2022). Our hospital has a high addressability for children with T1DM, being the only center admitting new T1DM pediatric cases from an area inhabited by approximately 1 million children. The criteria used for the diagnosis of T1DM included (1) fasting glucose values >126 mg/dL; (2) symptoms of hyperglycemia (polyuria, polydipsia, and unexplained weight loss) associated with a plasma glucose level >200 mg/dL independent of meal time; or (3) an oral glucose tolerance test with a 2 h plasma glucose level >200 mg/dL. The characteristics of our study group (2021 group) were compared with those of two other groups of patients diagnosed with T1DM in our clinic between March 2020 and February 2021 (2020 group) and in the period 2003–2019 (pre-pandemic group). All patients diagnosed in our hospital during the mentioned periods were included in the corresponding groups. Patients with other types of diabetes were excluded.

### 4.2. Variables

The data were extracted from the hospital’s digital records and included sex, age at diagnosis, living area, blood gas parameters, presence of SARS-CoV-2 antibodies, presence of T1DM-associated autoantibodies (GADA, ICA, and IA-2A), anti-thyroid peroxidase antibodies, anti-tissue transglutaminase antibodies, glycosylated hemoglobin levels, 25-OH vitamin D levels, personal history of SARS-CoV-2 infection, and a family history of type 1 diabetes. Positive GADA levels were considered those higher than 10 UI/mL, positive ICA levels were considered those higher than 0.9 UI/mL, and positive IA-2A levels were considered those higher than 10 UI/mL. The positive ATPO threshold was at 26 UI/mL. IgA or IgG anti-tissue transglutaminase antibodies had cut-off levels above 30 UI/mL. SARS-CoV-2 antibodies and islet autoantibodies were not routinely determined before April 2022; therefore, these data were only analyzed in the 2021 group of patients.

DKA was defined as a plasma pH value at the time of presentation below 7.3. The severity of DKA was established according to the 2018 International Society for Pediatric and Adolescent Diabetes (ISPAD) guidelines: pH < 7.1—severe DKA; pH < 7.2—moderate DKA; pH < 7.3—mild DKA. The normal range for HbA1c levels was 4.8–6.4%, and it was determined using an immunoturbidimetric method (Diabetes Control and Complications Trial standardized and National Glycohemoglobin Standardization Program certified).

### 4.3. Statistical Analysis

Categorical variables were reported as absolute and relative frequencies. Means and standard deviations were used for describing normally distributed continuous variables, and medians and interquartile ranges (IQR) were used for non-normally distributed continuous variables. The normal distribution of variables was determined using Kolmogorov–Smirnov and Shapiro–Wilks tests. Patient characteristics were compared between subgroups of our study sample according to (1) the presence of SARS-CoV-2 antibodies and (2) the presence of a DKA diagnosis at T1DM onset. The entire group was then compared to the group of patients diagnosed between March 2020 and February 2021 (2020 group) and, separately, to the group of patients diagnosed in the period 2003–2019 (pre-pandemic group). Differences between compared (sub)groups were tested using χ^2^ tests for categorical variables, *t*-tests for normally distributed continuous variables, and Mann–Whitney U tests for non-normally distributed continuous variables. A two-tailed *p*-value of 0.05 was considered statistically significant. The data collection and statistical analysis were performed using Microsoft Excel 16.0 (Microsoft Corp. S.R.L., MI, USA) and SPSS 29.0 (IBM Corp., New York, NY, USA).

## 5. Conclusions

Children with a positive SARS-CoV-2 serology presented positive levels of IA-2A antibodies more frequently. Moreover, in the same group, higher percentages of patients tested positive for all three analyzed antibodies, for both IA-2A and GADA, and both IA-2A and ICA. No difference in the percentage of patients presenting with DKA was found between the groups with positive and negative SARS-CoV-2 antibodies. In the group of patients who tested positive for SARS-CoV-2 antibodies, HbA1c levels were higher, and males were better represented.

When we divided the study sample according to the presence or absence of DKA at T1DM onset, no difference between the groups was identified regarding the positivity rates of SARS-CoV-2 and islet antibodies. The only difference identified was a lower C-peptide level in the patients diagnosed with DKA.

The patients diagnosed between April 2021 and April 2022 presented DKA and its severe form more frequently and had higher HbA1c levels compared to the patients diagnosed before the pandemic, while there was no difference in these factors between the first group of patients and children diagnosed in the period March 2020–February 2021. The children from the 2021 group had a higher age of diagnosis than the children from the other two groups.

## Figures and Tables

**Table 1:** Characteristics of the groups of patients with positive and negative SARS-CoV-2 serology.

Characteristics	SARS-CoV-2 Positive Antibodies N = 37	SARS-CoV-2 Negative Antibodies N = 121	*p*-Values
Sex			<0.001
Female	16 (43.2%)	67 (55.4%)	
Male	21 (56.8%)	54 (44.6%)	
Background			0.40
Rural	15 (40.5%)	40 (33.1%)	
Urban	22 (59.5%)	81 (66.9%)	
Age of onset (mean, SD)	8.18 ± 3.58	8.53 ± 4.62	0.63
pH (median, (IQR))	7.26 (7.11–7.40)	7.25 (7.17–7.40)	0.73
DKA			0.45
Present	26 (72.2%)	74 (65.5%)	
Absent	10 (27.8%)	39 (34.5%)	
Severe DKA			0.81
Present	8 (22.2%)	23 (20.4%)	
Absent	28 (77.8%)	90 (79.6%)	
HbA1c (mean, SD)	12.96 ± 1.97%	11.88 ± 1.90%	0.004
GADA			0.31
Present	22 (62.9%)	84 (71.8%)	
Absent	13 (37.1%)	33 (28.2%)	
ICA			0.89
Present	19 (54.3%)	62 (53.0%)	
Absent	16 (45.7%)	55 (47.0%)	
IA-2A			0.01
Present	25 (73.5%)	58 (50.4%)	
Absent	9 (26.5%)	57 (49.6%)	
GADA + ICA + IA-2A			0.03
Present	14 (41.2%)	27 (23.3%)	
Absent	20 (58.8%)	89 (76.7%)	
GADA + ICA			0.91
Present	15 (42.9%)	49 (41.9%)	
Absent	20 (57.1%)	68 (58.1%)	
GADA + IA-2A			0.03
Present	19 (55.9%)	41 (35.7%)	
Absent	15 (40.5%)	74 (64.3%)	
ICA + IA-2A			0.02
Present	18 (52.9%)	37 (32.2%)	
Absent	16 (43.2%)	78 (67.8%)	
Vitamin D (median, IQR, ng/mL)	23.30 (15.30–30.02)	25.10 (15.75–30.40)	0.75
C-peptide (median, IQR, ng/mL)	0.58 (0.42–1.02)	0.57 (0.37–1.01)	0.82

*p*-values resulted from χ^2^ statistics for categorical variables, *t*-tests for normally distributed continuous variables, and Mann–Whitney U tests for non-normally distributed continuous variables. The null hypothesis of no difference between the groups was tested.

**Table 2:** The characteristics of the groups of patients with and without a DKA diagnosis at T1DM onset.

Characteristics	DKA N = 100	Non-DKA N = 49	*p*-Values
Sex			0.72
Female	50 (50.0%)	26 (53.1%)	
Male	50 (50.0%)	23 (46.9%)	
Background			0.15
Rural	31 (31.0%)	21 (42.90%)	
Urban	69 (69.0%)	28 (57.10%)	
Age of onset (median, IQR)	7.00 (4.00–11.00)	11.00 (7.50–12.00)	0.04
SARS-CoV-2 antibodies			0.45
Present	26 (26.0%)	10 (20.4%)	
Absent	74 (74.0%)	39 (79.6%)	
HbA1c (mean, SD)	12.34 ± 1.89%	11.92 ± 2.13%	0.22
GADA			0.79
Present	66 (68.8%)	34 (70.8%)	
Absent	30 (31.2%)	14 (29.2%)	
ICA			0.34
Present	54 (56.3%)	23 (47.9%)	
Absent	42 (43.8%)	25 (52.1%)	
IA-2A			0.08
Present	57 (61.3%)	22 (45.8%)	
Absent	36 (38.7%)	26 (54.2%)	
GADA + ICA + IA-2A			0.95
Present	25 (26.6%)	13 (27.1%)	
Absent	69 (73.4%)	35 (72.9%)	
GADA + ICA			1.00
Present	40 (41.7%)	20 (41.7%)	
Absent	56 (58.3%)	28 (58.3%)	
GADA + IA-2A			0.21
Present	41 (44.1%)	16 (33.3%)	
Absent	52 (55.9%)	32 (66.7%)	
ICA + IA-2A			0.53
Present	36 (38.7%)	16 (66.7%)	
Absent	57 (61.3%)	32 (33.3%)	
Vitamin D (median, IQR, ng/mL)	23.95 (14.37–30.37)	25.80 (17.25–30.45)	0.57
C-peptide (median, IQR, ng/mL)	0.49 (0.33–0.85)	0.78 (0.51–1.16)	<0.001

*p* values resulted from χ^2^ statistics for categorical variables, *t*-tests for normally distributed continuous variables, and Mann–Whitney U tests for non-normally distributed continuous variables. The null hypothesis of no difference between the groups was tested.

**Table 3:** The characteristics of the groups of patients diagnosed in April 2021–April 2022 vs. March 2020–February 2021 vs. before the COVID-19 pandemic.

Characteristics	2021 Group N = 158	2020 Group N = 147	*p*-Values 2021 Group vs. 2020 Group	Pre-Pandemic Group N = 312	*p*-Values 2021 Group vs. Pre-Pandemic Group
Gender			0.53		0.15
Female	83 (52.5%)	72 (48.9%)		142 (45.5%)	
Male	75 (47.5%)	75 (51.0%)		170 (54.5%)	
Background			0.55		<0.001
Rural	55 (34.8%)	56 (38.1%)		50 (16.0%)	
Urban	103 (65.2%)	91 (61.9%)		262 (84.0%)	
Age of onset	9.00	7.20	<0.01	7.00 (3.00–10.00)	<0.01
(median, IQR)	(5.00–12.00)	(7.07–7.30)			
DKA			0.83		<0.001
Present	100 (67.1%)	97 (65.9%)		123 (39.4%)	
Absent	49 (32.9%)	50 (34.0%)		189 (60.6%)	
Severe DKA			0.15		0.002
Present	31 (20.8%)	41 (27.9%)		33 (10.5%)	
Absent	118 (79.2%)	106 (72.1%)		279 (89.5%)	
HbA1c	12.14% ± 1.96%	12.47% ± 2.19	0.18	11.32% ± 2.18%	<0.001
(mean, SD)					

*p* values resulted from χ^2^ statistics for categorical variables, *t*-tests for normally distributed continuous variables, and Mann–Whitney U tests for non-normally distributed continuous variables. The null hypothesis of no difference between the groups was tested.

## Data Availability

Not applicable.
